# Supplementary material for: The use of technical replication for detection of low-level somatic mutations in next-generation sequencing
Source: Nat Commun. 2019 Mar 5;10:1047. doi: 10.1038/s41467-019-09026-y (PMC6400950; doi:10.1038/s41467-019-09026-y)
Supplement: Supplementary file 1 — Supplementary Information [file 41467_2019_9026_MOESM1_ESM.pdf]

Supplementary Information

The use of technical replication for detection of low-level somatic mutations in next-generation sequencing

Junho Kim[1], Dachan Kim[1], Jae Seok Lim[2], Ju Heon Maeng[1], Hyeonju Son[1], Hoon-Chul Kang[3], Hojung Nam[4], Jeong Ho Lee[2,*] and Sangwoo Kim[1,*]

[1]Department of Biomedical Systems Informatics and Brain Korea 21 PLUS Project for Medical Science, Yonsei University College of Medicine, Seoul 03722, South Korea
[2]Graduate School of Medical Science and Engineering, KAIST, Daejeon 34141, South Korea
[3]Division of Pediatric Neurology, Department of Pediatrics, Pediatric Epilepsy Clinics, Severance Children's Hospital, Epilepsy Research Institute, Yonsei University College of Medicine, Seoul 03722, South Korea
[4]School of Electrical Engineering and Computer Science, Gwangju Institute of Science and Technology, Gwangju 61005, South Korea

*Corresponding author

Sangwoo Kim, Ph.D.
Assistant Professor,
Dept. of Biomedical Systems Informatics, Yonsei University College of Medicine, Seoul 03722, Korea
+82-2-2228-0913 (office), +82-2-2227-8129 (fax),
E-mail: swkim@yuhs.ac
Homepage: http://www.tgilab.org/
ORCID ID: 0000-0001-5356-0827

Jeong Ho Lee, M.D., Ph.D.
Associate Professor,
Graduate School of Medical Science and Engineering, Korea Advanced Institute of Science and Technology (KAIST), Korea+82-42-350-4246 (office),
E-mail: jhlee4246@kaist.ac.kr
Homepage: https://tnl.kaist.ac.kr

Contents

Supplementary Methods 2
Basic preprocessing of NGS data 2
Parameter adjustment of variant calling algorithms 2
Filtration of variant candidates in the RePlow model 4
The benchmarks for the conventional post-filtering steps 5
RePlow command line used in the analysis 6
Supplementary Figures 7
Supplementary Tables 24

List of Supplementary Figures

1. Sensitivity and FPR of conventional callers by sequencing depth and VAF
2. Sensitivity and FPR of conventional callers by sequencing depth and VAF after parameter adjustment
3. Rejected ratio of true variant by triallelic site
4. Receiver-operating characteristic (ROC) curves for the classification of true and false positive calls from the test-base data of sample B (1% VAF)
5. The benchmarks of conventional post-filtering steps from the test-base data (sample B, 1% VAF)
6. Sensitivity and FPR of primitive approaches with sample A (0.5% VAF) for each platform
7. The fraction of non-variant sites with the false positive call for all depths and platforms
8. Increased number of triallelic rejection at non-variant sites
9. Comparison of VAF concordance between true and false positive calls
10. Performance assessment with the library duplicates of test-base data (sample A, 0.5% VAF)
11. Performance assessment with the single and duplicate libraries for the same depth of coverage (sample B, 1% VAF)
12. Performance assessment with the library triplicates of test-base data (sample B, 1% VAF)
13. Performance assessment with the library triplicates of test-base data (sample A, 0.5% VAF)
14. Experimental validation of low-level mutations from the samples negative for pathogenic mutations in previous analysis
15. Performance assessment of RePlow with the typical mutation rate (3×10^{-6}) for the library duplicates of sample B (1% VAF).

List of Supplementary Tables

1. Summary of the test-base sequencing data and read-depth information
2. Summary of the reference standard sequencing data and read-depth information

Supplementary Methods

Basic preprocessing of NGS data

BWA-MEM[1] (http://bio-bwa.sourceforge.net/) was used to align raw sequence reads to the reference genome for both types of Illumina sequencing data (ILH and ILA). Read sorting and indexing were performed by Picard (http://broadinstitute.github.io/picard/) for all BAM files. Marking PCR duplicates was applied only to the data from ILH. Indel realignment of Genome Analysis Tool Kit[2] (GATK, https://software.broadinstitute.org/gatk/) was finally applied to make analysis-ready mapped data. We followed the pipeline of OTG-snpcaller[3] to preprocess data from ITA. TMAP (https://github.com/iontorrent/TMAP/) was used for sequence read alignment. Removing duplicates according to the alignment score tag (RDAST) and alignment optimize structure (AOS) processes were carried out following the workflow of OTG-snpcaller. Indel realignment of GATK was identically applied for ITA data as a final step.

Parameter adjustment of variant calling algorithms

Eight independent callers were tested to detect variants from the mixture datasets: MuTect, VarScan2, Strelka, VarDict, FreeBayes, Virmid, LoFreq, and deepSNV. Since all somatic callers had difficulties with detecting mutations from sample A and B (0.5% and 1% VAF) with their default settings, we adjusted parameters to obtain better performance. Besides each caller-specific options, following three criteria were commonly applied for adjustments: 1) if a given caller requires a proportion of disease cell population (e.g. tumor purity/cellularity, contamination level of normal cell, expected VAF), we provided exact value for each mixture sample to achieve the best performance (1% cellularity (0.5% VAF) for sample A, 2% cellularity (1% VAF) for sample B, 10% cellularity (5% VAF) for sample C, and 20% cellularity (10% VAF) for sample D). 2) if a given caller accepts the threshold for minimum allele frequency, we provided 0.25% (a half of true VAF from sample A) to remove low-level background noises without affecting true mutation detection. 3) For amplicon datasets (ILA and ITA), we disabled filters for strand bias and clustered positions due to the nature of their generation. Specific settings used for each caller are described as follows. Despite these individual parameter adjustments, all callers were failed to discriminate true low-VAF mutations (0.5% and 1%) from background errors; all adjusted calls that achieved fair sensitivity for sample A and B were accompanied with enormous false positive rates.

MuTect[4]: v1.1.7 was used for all analyses in this study. Three count-based thresholds (-dcov, --max_alt_alleles_in_normal_count, and –max_alt_alleles_in_normal_qscore_sum) were disabled to discard unsuitable limit for high-depth data. Fraction-based thresholds (e.g. –max_alt_alleles_in_normal_fraction) were kept with default values to maintain the intended uses. --fraction_contamination and --tumor_f_pretest were set to 0 to disable those filters, which prevent variant calls with low-frequency

because of the possibility of contamination or computational efficiency. For ILA and ITA datasets, four filters related with strand bias and clustered positions including --strand_artifact_lod, --pir_median_threshold, --pir_mad_threshold, and --gap_events_threshold were additionally disabled due to the natural property of amplicon data. dbSNP filter was not applied because most of the true variants in test-base and reference standards are dbSNP entries. Calls with 'KEEP' judgement were considered as final somatic candidates.

VarScan2[5]: v2.3.9 was used with samtools v0.1.19 that generates mpileup files for input data. Coverage limit was disabled (-d) to cover the entire high-depth information. Tumor purity (--tumor-purity) was adjusted from 0.01 (sample A) to 0.2 (sample D) for all datasets based on the true cellularity of each mixture. VAF thresholds (--min-var-freq) was set to 0.0025, to reduce background errors without affecting true mutation detection from sample A. Unlike other callers, VarScan2 was evaluated with raw SNV call results rather than high-confidence set, because its high-confidence filter (processSomatic) is fixed to filter out the variants with VAF<0.1.

Strelka[6]: v1.0.14 was used to call SNV candidates. Depth filter was turned off by adjusting related parameters (isSkipDepthFilters and maxInputDepth). For amplicon datasets, ssnvNoiseStrandBiasFrac was adjusted to zero to disable strand bias filtering. To detect mutations with low-frequency (≤1% VAF), ssnvNoise and sindelNoise values in the configuration option were lowered up to 1000-fold but hardly showed any improvements for their detection. Calls from the passed.somatic.snvs.vcf were considered as final somatic candidates.

Vardict[7]: Paired variant calling mode was applied to all three platforms by using var2vcf_paired.pl. Minimum allele frequency (-f) was set to 0.0025 to remove error calls without affecting mutation detection from sample A. For ILA and ITA datasets, amplicon mode calling of Vardict was additionally tested, which filters out candidates partially observed within targeted amplicons. Customized BED files that includes the genomic location information of PCR primer and insert were prepared to apply this mode. Only variant calls with 'PASS' tag at the FILTER field are considered as somatic candidates for both modes.

FreeBayes: v1.1.0 was used to call SNV candidates. Paired variant calling was performed by applying both of disease and normal BAM files simultaneously. --pooled-continuous option was used to get all results regardless of genotype model. Minimum alternative fraction (-F) was set to 0.0025 to remove error calls without affecting mutation detection from sample A.

Virmid[8]: v1.1.1 was used to call SNV candidates. Depth filter was disabled with the option -M -1. We provided exact value of alpha (proportion of control sample, 0.99, 0.98, 0.9, and 0.8 for sample A, B, C, and D, respectively) rather than used the estimated value to obtain the best detecting performance. Calls from

the virmid.som.passed.vcf were considered as final somatic candidates.

LoFreq[9]: v2.1.2 was used with the somatic mode. The option --tumor-mtc was set to FDR to get more sensitive calls. Although the use of dbSNP information is highly recommended by the author, we didn't use this filter because most of the true variants in test-base and reference standards are dbSNP entries. Calls from the somatic_final.snvs.vcf were considered as final somatic candidates.

deepSNV[10]: v1.22.0 was installed through the deepSNV Bioconductor package. Benjamini-Hochberg correction with the significance level of 0.05 was used for multiple testing to get more sensitive calls. The entire calls were considered as final somatic candidates.

Filtration of variant candidates in the RePlow model

Although a given position is called as a variant by RePlow model, it can still be a false positive due to various causes including mapping ambiguity, indel proximity, or misclassification of germline variants. To reduce them, we applied several filtering criteria that have been commonly used for conventional methods. Two different categories of filters are designed, position quality filter and data quality filter, which removes false positives arisen from the genomic complexity of target location or from the poor quality of sequenced data, respectively. Most filters adopt fraction- or probability-based thresholds rather than count-based ones, for general use with high-depth sequencing data. Lenient thresholds were applied as default settings compared with conventional methods, to create a universal model which is not platform-dependent.

Position quality filter removes variant candidates if at least one sequencing data corresponds to the following criteria:

1. Low mapping quality: if overall ratio of ambiguously mapped reads (MQ<20) exceeds a half of sequencing depth at the corresponding site

2. Indel proximity: if more than 50% of the mapped reads possess indels located within 3 bp from the corresponding site

3. Observed in control: if variant alleles are observed at the corresponding site of matched control data and the probability that an error will cause a larger VAF is less than 0.01 ($P(X > x_{BE} | M_0) < 0.01$)

4. Homopolymeric artifact: If four or more identical reference bases are continuously connected from the corresponding site and the variant allele is the same as the first inconsistent base next to them

For data quality filter, eight criteria are adopted as follows. Criteria 7 and 8 are applied if more than half of replicates are corresponding to the same criteria.

1. Low mapping quality: if more than half of reads with the B allele are ambiguously mapped

(MQ<20)

2. Indel proximity: if more than 40% of the B alleles are located within 3 bp from indels

3. Excessive number of mismatches: if more than 30% of reads with B allele possess more than 5% of the read length as mismatch

4. Clustered position: if more than 80% of the B alleles are located within 3 bp from the end of read

5. Successive mutation: if more than 80% of the B alleles are successively located with another mismatch

6. Read-pair orientation bias: if more than 95% of reads with B allele are skewed to one read-pair orientation and reads with reference allele are not

7. Low base quality: if more than 50% of the B alleles have poor base quality scores (BQ<15) or expected count of background error is less than a half of B allele count ($b_{BE}{}^{i}{}_{j} < b^{i}{}_{j} / 2$)

8. Variant-free fraction: if the B allele is not observed in more than half of replicates

Note that there is no filter for the triallelic site, because RePlow performs analysis by substitution type rather than position; RePlow separately assesses each substitution type for a given position. This approach is especially effective when additional background errors occur in a variant site, which is common in high-depth sequencing data (Supplementary Fig. 2). All threshold values of each filter can be adjusted by a user in a practical use.

The benchmarks for the conventional post-filtering steps

Most modern sequence analysis pipelines have included additional post-filtering steps after variant calling to reduce many systematic errors, such as OxoG or FFPE artifacts. Despite the great false positive reduction in RePlow, it may raise concern that such improvements will also be achieved by conventional post-filtering steps. In order to prove that false positives cannot be removed by conventional filters to the same dramatic extent as with our method, we have applied four post-filtering steps to the results of MuTect and compared them to our results (Supplementary Fig. 5). Followings are the detailed description of the used external filters.

Panel of Normals (PoN): The PoN filter has been used to remove germline variants and systematic artifacts that commonly arise from particular platforms or experimental procedures. Since there is no publically available PoN, we constructed our own PoN using 2,520 whole-exome sequencing data from the 1000 Genomes project, following the instruction from the GATK best practice workflow to remove every variant that has been detected in ≥2 normal individuals. When we apply the PoN filter to our test data sets, we exempted all true positive sites from the PoN filter because all true sites in our spike-in data sets

are common SNPs that are easily filtered out by PoN. This makes the PoN filter suffers no sensitivity loss in the benchmark, which may show the most optimistic results by removing the risk of using PoN at all.

Read-pair orientation filter: Specific type of artifacts such as OxoG or FFPE artifacts are well-known to have a significant read-orientation bias based on their source of origin[11,12]. Previous study[12] has deeply analyzed the pattern of these artifacts and developed a trained model to eliminate them considering the measured read-pair orientation bias and LOD_T values from the MuTect call. We utilized the same trained model and threshold in the previous work to remove the biased artifacts:

$LOD_T > -10+(100/3)\times FoxoG$

FFPE and low-confidence filters: We adopted two computational filters from the ngs-filter modules developed by Memorial Sloan Kettering Cancer Center (https://github.com/mskcc/ngs-filters). Since FFPE artifacts are well-known to be easily generated at the CpG sites (NCG>NTG), the FFPE filter eliminates the candidates within this context if the allele fraction is less than 0.1. The low-confidence filter removes the candidate if it fulfills either one of followings: i) alternative count > 1 in the matched normal, ii) total depth < 20 in the disease sample, iii) alternative count < 4 in the disease sample.

RePlow command line used in the analysis

All RePlow results were obtained by version 1.1 with the default parameter settings regardless of sequencing platform, with the sole exception of mutation rate for the test-base data sets as we have described (see *Assessment of performance with replicates* in Methods). We have used the mutation rate of 8.05×10^{-3}, 8.60×10^{-3}, and 4.83×10^{-3} for ILH, ILA, and ITA with the test-base data sets. For the independent validation data set (commercial reference standard sequenced using cancer panels) and the epilepsy patient data, a default mutation rate (3×10^{-6}) was applied for all samples. The following is a RePlow command line template that used in the analysis:

```
java -jar RePlow.jar \
        -r <reference_genome.fa> \
        -b <rep1.bam, rep2.bam> \
        -N <normal.bam> \
        -T <target_region.bed> \
        -v <GATK_germline_call_from_normal_bam.vcf> \
        -R <Rscript_path> \
        -o <output_dir_path> \
        -m <mutation_rate>
```

Supplementary Figures

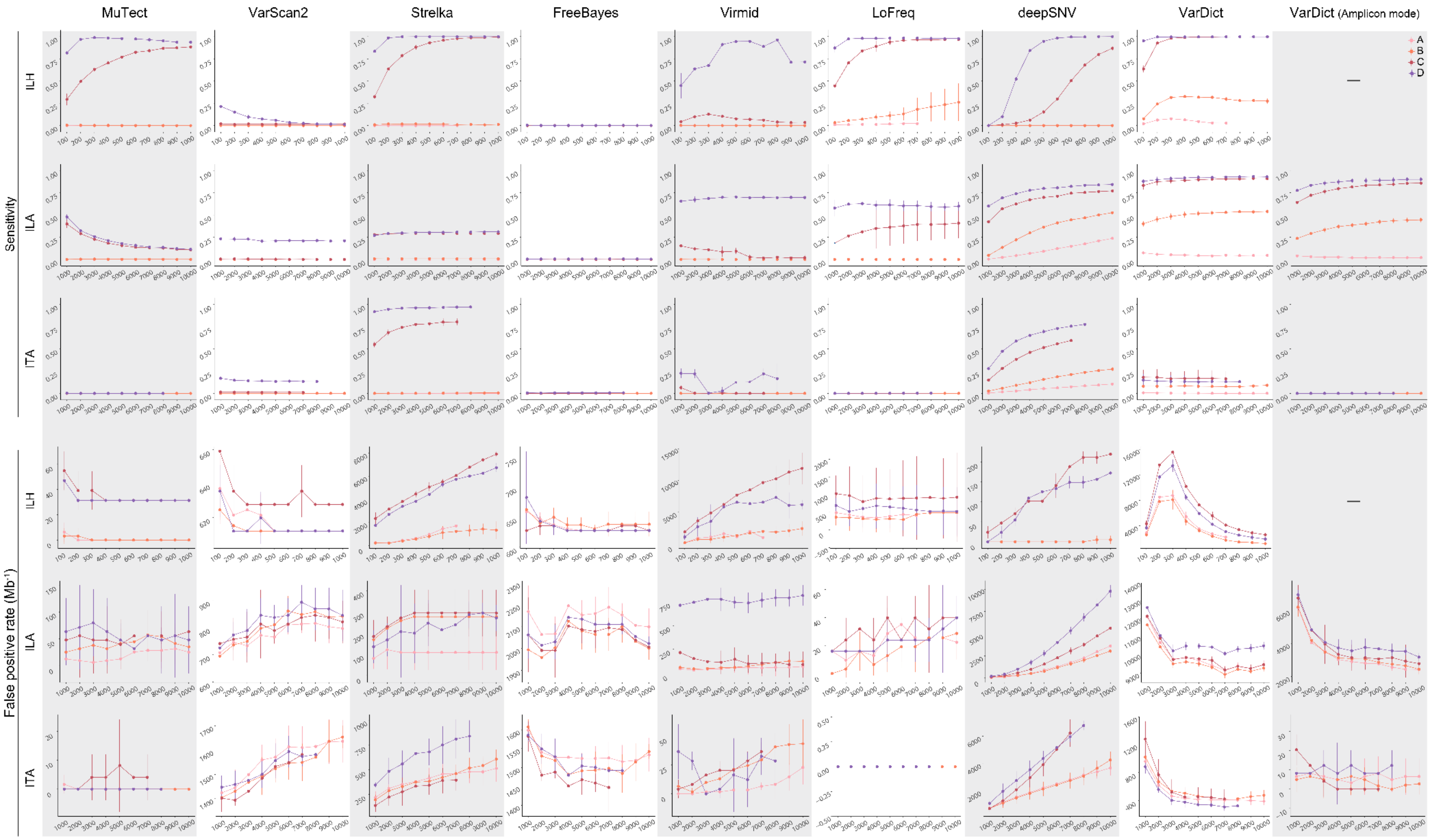

Supplementary Figure 1. Sensitivity and FPR of conventional callers by sequencing depth and VAF. Performances for the near default setting (disabled coverage limit) of MuTect, VarScan2, Strelka, FreeBayes, Virmid, LoFreq, deepSNV, and VarDict are depicted. The vast majority of callers lost most of the true mutations in sample A and B (VAF≤1%) regardless of their coverage. Error bars, 95% confidence interval. Source data are provided as a Source Data file.

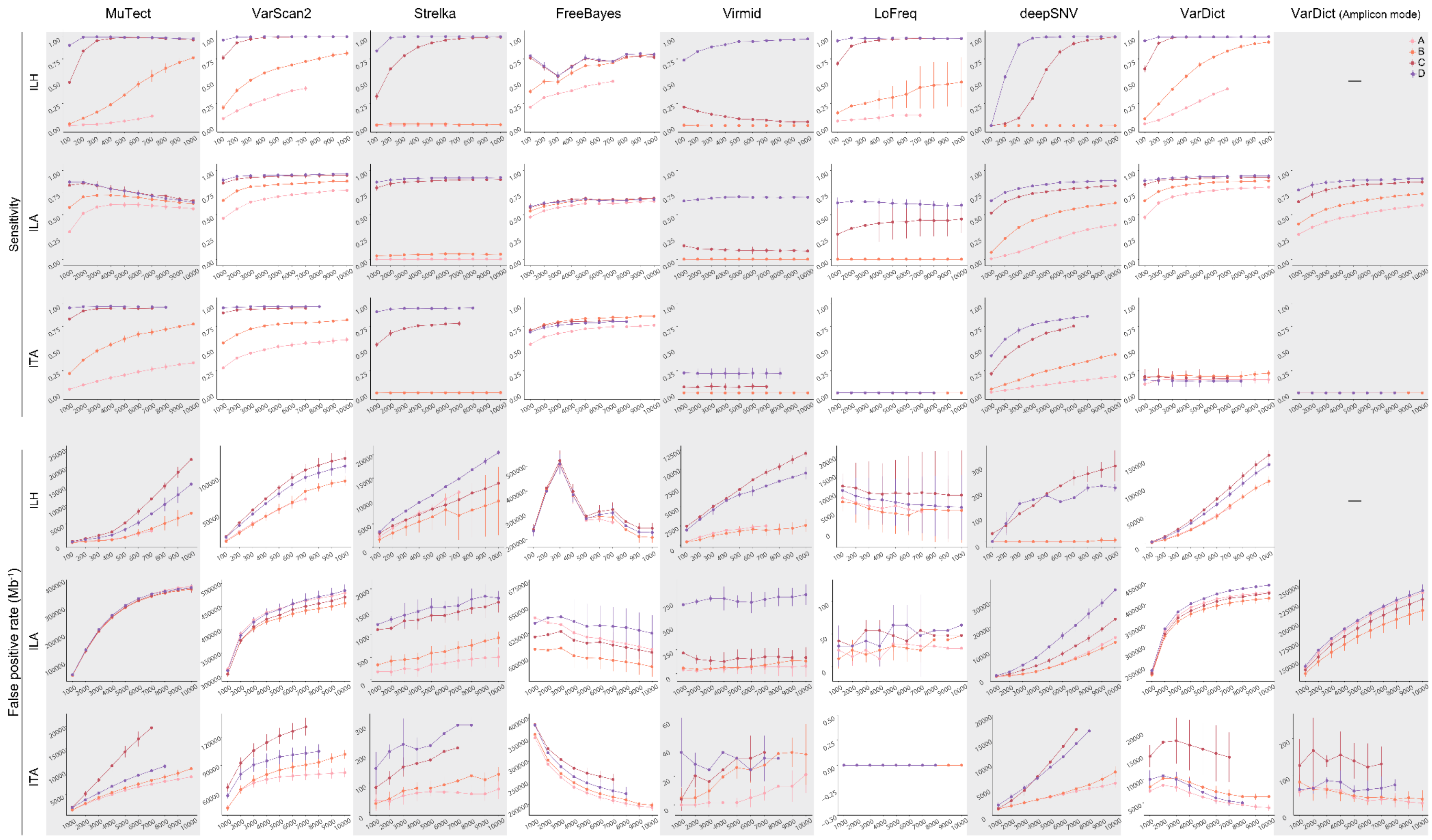

Supplementary Figure 2. Sensitivity and FPR of conventional callers by sequencing depth and VAF after parameter adjustment. Performances with adjusted

parameters are depicted for all callers. Parameter adjustment rescued low-level mutations in sample A and B for many callers, but an explosion of FPR were inevitably followed. Detailed parameter settings for each caller are described in Supplementary Methods. Error bars, 95% confidence interval. Source data are provided as a Source Data file.

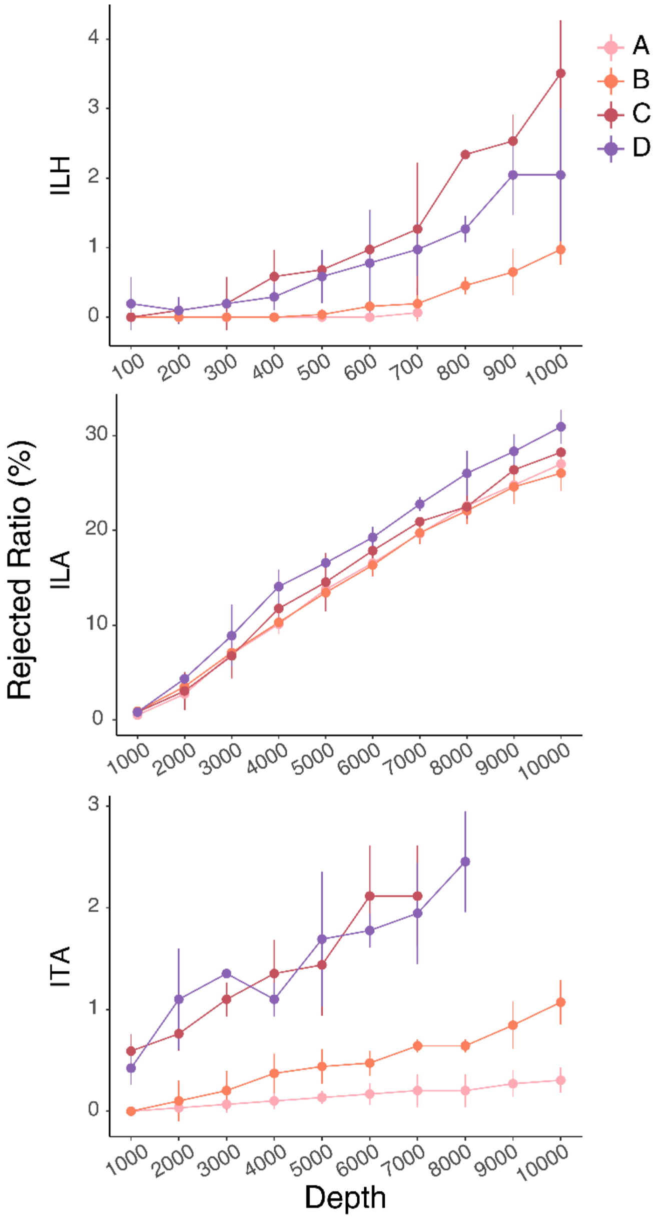

Supplementary Figure 3. Rejected ratio of true variant by triallelic site. In high-depth sequencing data, accumulated errors at true variant sites hinder the mutation detection by inducing the triallelic-like signals. At 10,000x coverage in ILA, about 30% of true variants are detected but rejected by the triallelic filter of MuTect, regardless of the VAF of mutations. Error bars, 95% confidence interval. Source data are provided as a Source Data file.

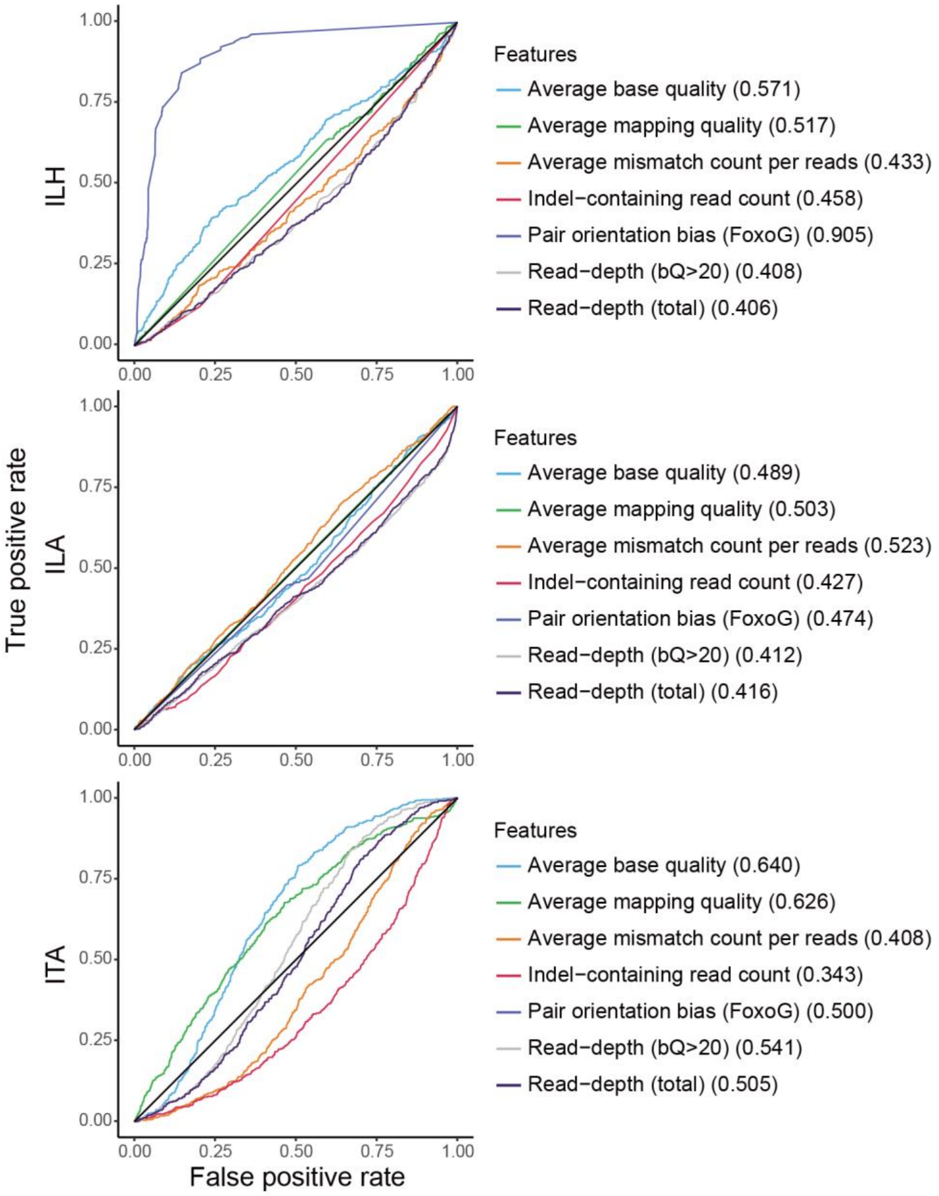

Supplementary Figure 4. Receiver-operating characteristic (ROC) curves for the classification of true and false positive calls from the test-base data of sample B (1% VAF). Seven sequencing features that are commonly used for the computational quality filters were selected and tested to classify true low-level variants from error calls. Except read-pair orientation bias in ILH, all features failed to discriminate true variants regardless of the sequencing platform. Note that read-pair orientation bias in ITA could not be calculated, because it is single-end data. The area under the ROC curve (AUC) is indicated for each feature. Features with the AUC less than 0.5 represent that false positive calls had better quality (e.g. higher base call quality, lower number of indel-containing read count, etc) than true mutations.

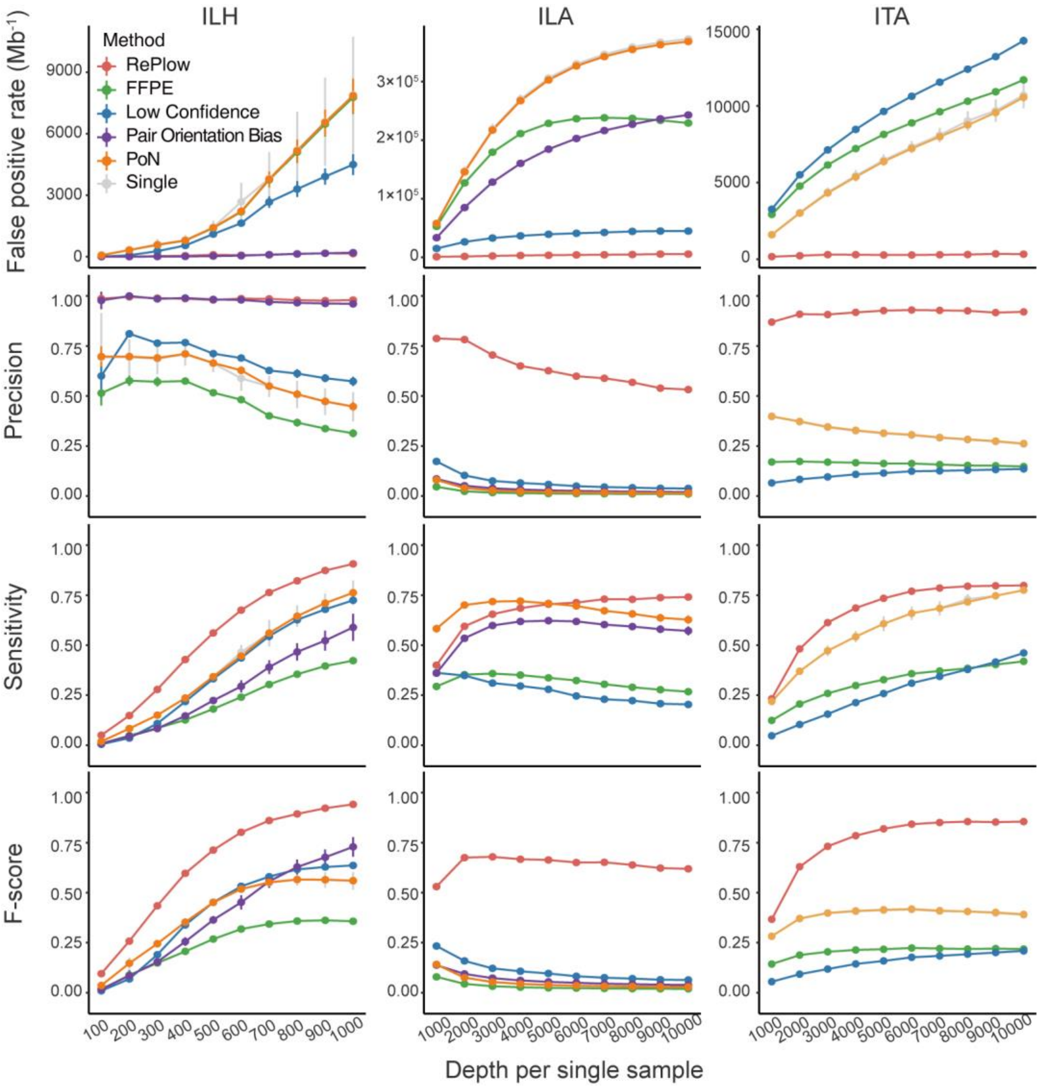

Supplementary Figure 5. The benchmarks of conventional post-filtering steps from the test-base data of sample B (1% VAF). Four conventional filters (Panel of Normals (PoN), read-pair orientation bias, FFPE, and low-confidence filters) were tested and compared to the results of our method (RePlow). Note that read-pair orientation filter could not be applied in ITA because it is single-end data. The detailed information of the used external filters is described in the Supplementary Methods. Source data are provided as a Source Data file.

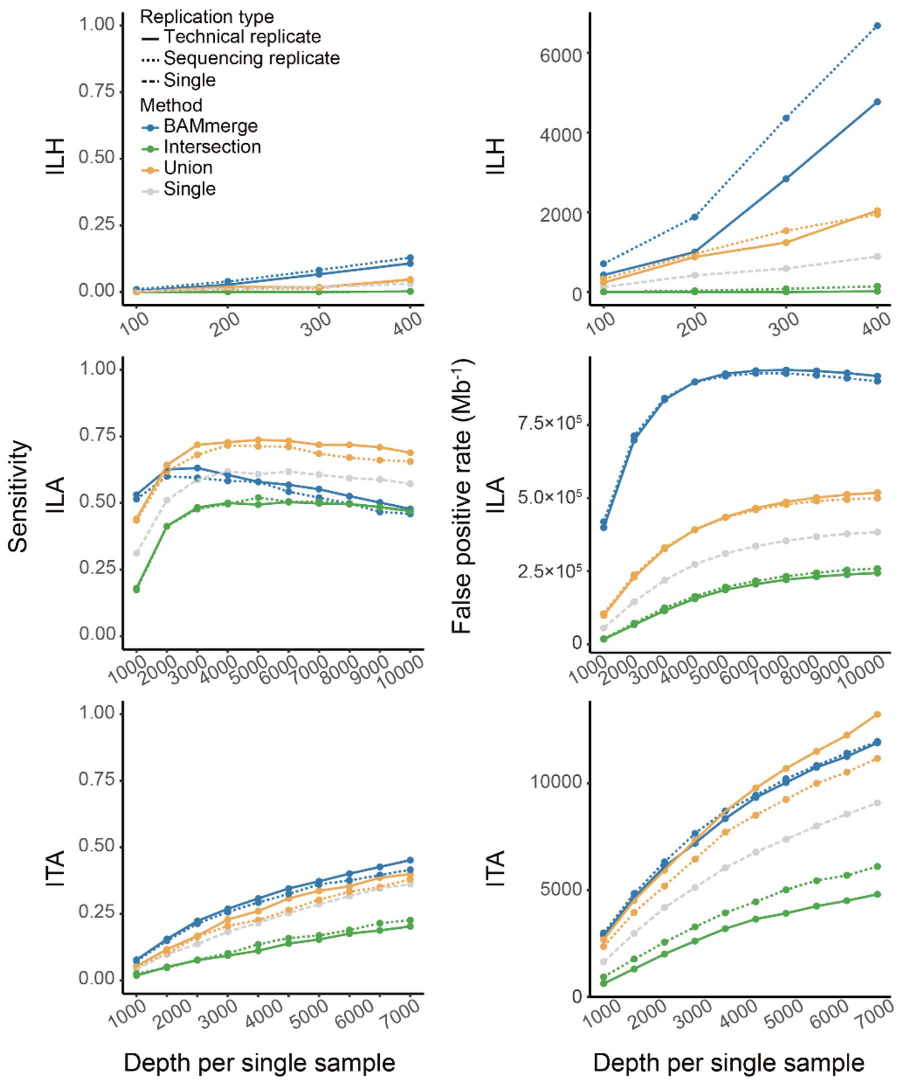

Supplementary Figure 6. Sensitivity and FPR of primitive approaches with sample A (0.5% VAF) for each platform. Primitive approaches were applied for both library (solid lines) and sequencing (dotted lines) duplicates. Calls from the single sample (dashed lines) are also depicted to evaluate the improvement with replicates. All mutation calls were made by MuTect. Source data are provided as a Source Data file.

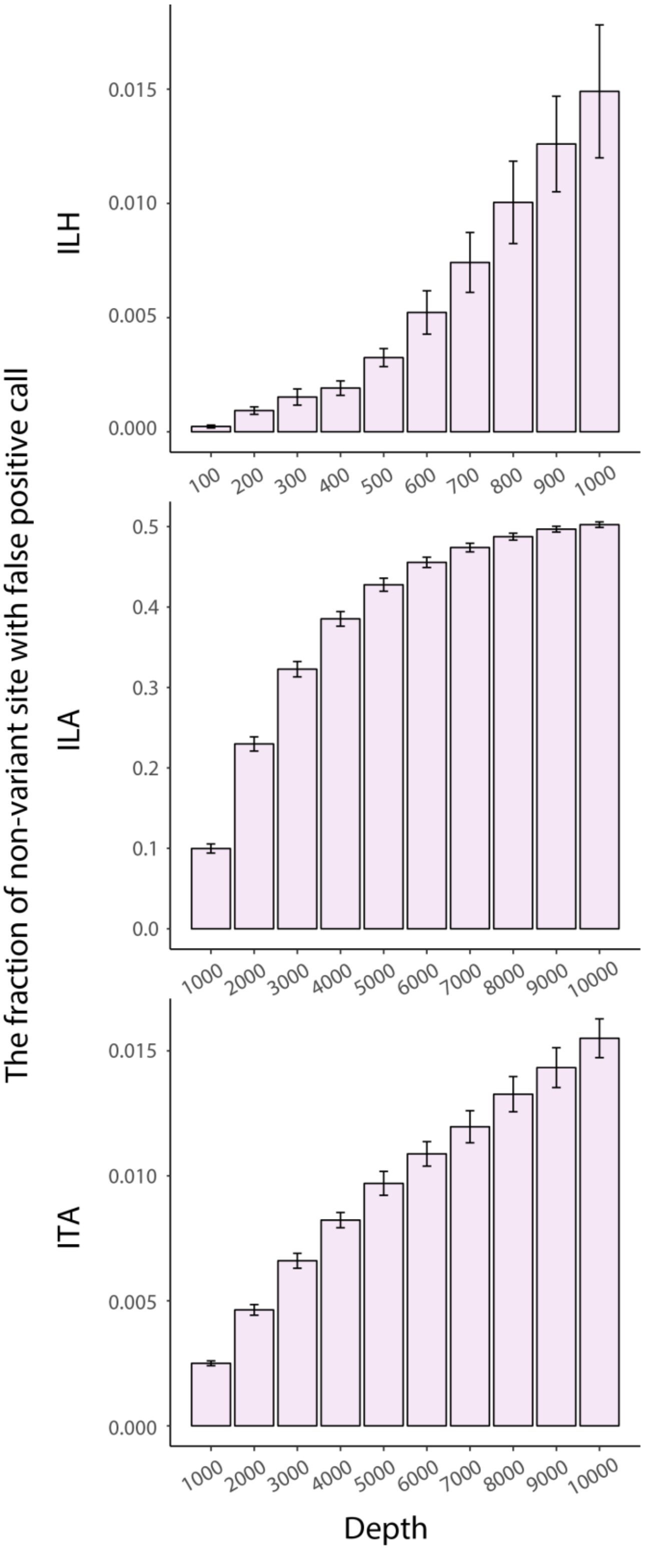

Supplementary Figure 7. The fraction of non-variant sites with the false positive call for all depths and platforms. The fraction is calculated by dividing the number of false positive call by the total size of target region except the true variant sites. The number of false positive calls at non-variant sites is increased in higher read-depths regardless of the sequencing platform. At 10,000x of ILA, 50% of non-variant sites were called by MuTect due to the excessive occurrence of background error. Source data are provided as a Source Data file.

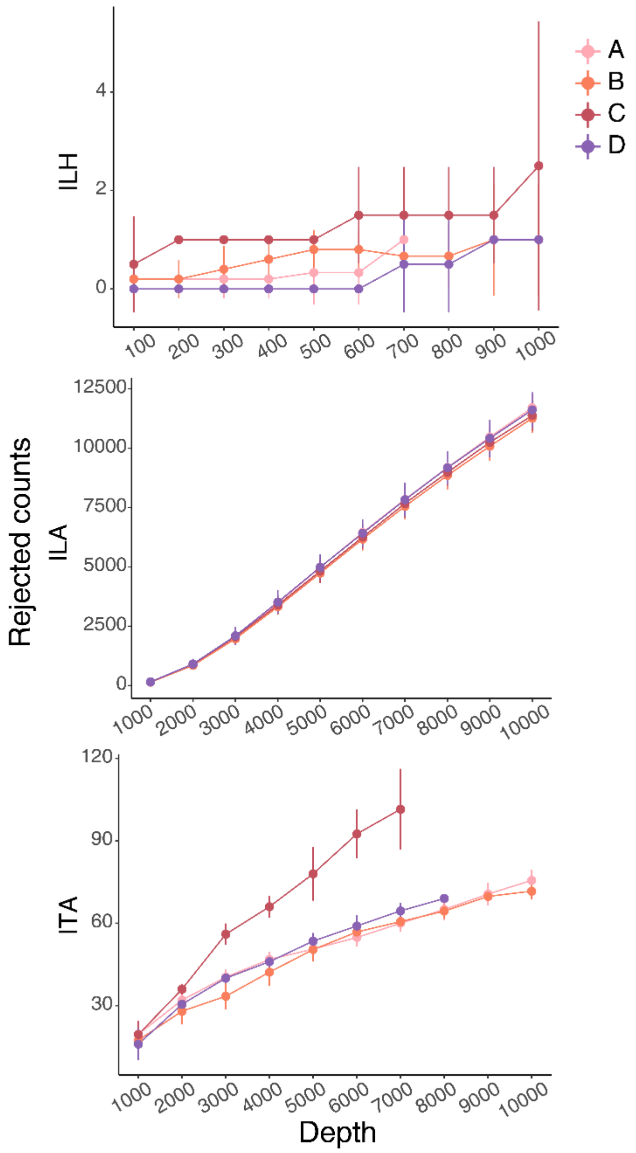

Supplementary Figure 8. Increased number of triallelic rejection at non-variant sites. Due to the increased occurrence of background error in higher depth, triallelic rejection is occurred even at non-variant sites according to the accumulation of more than two different erroneous alleles at the same position. Such rejection prevents making calls at non-variant sites and thus reduces overall false positive rate. In ILA, the number of triallelic rejection is linearly increased. Source data are provided as a Source Data file.

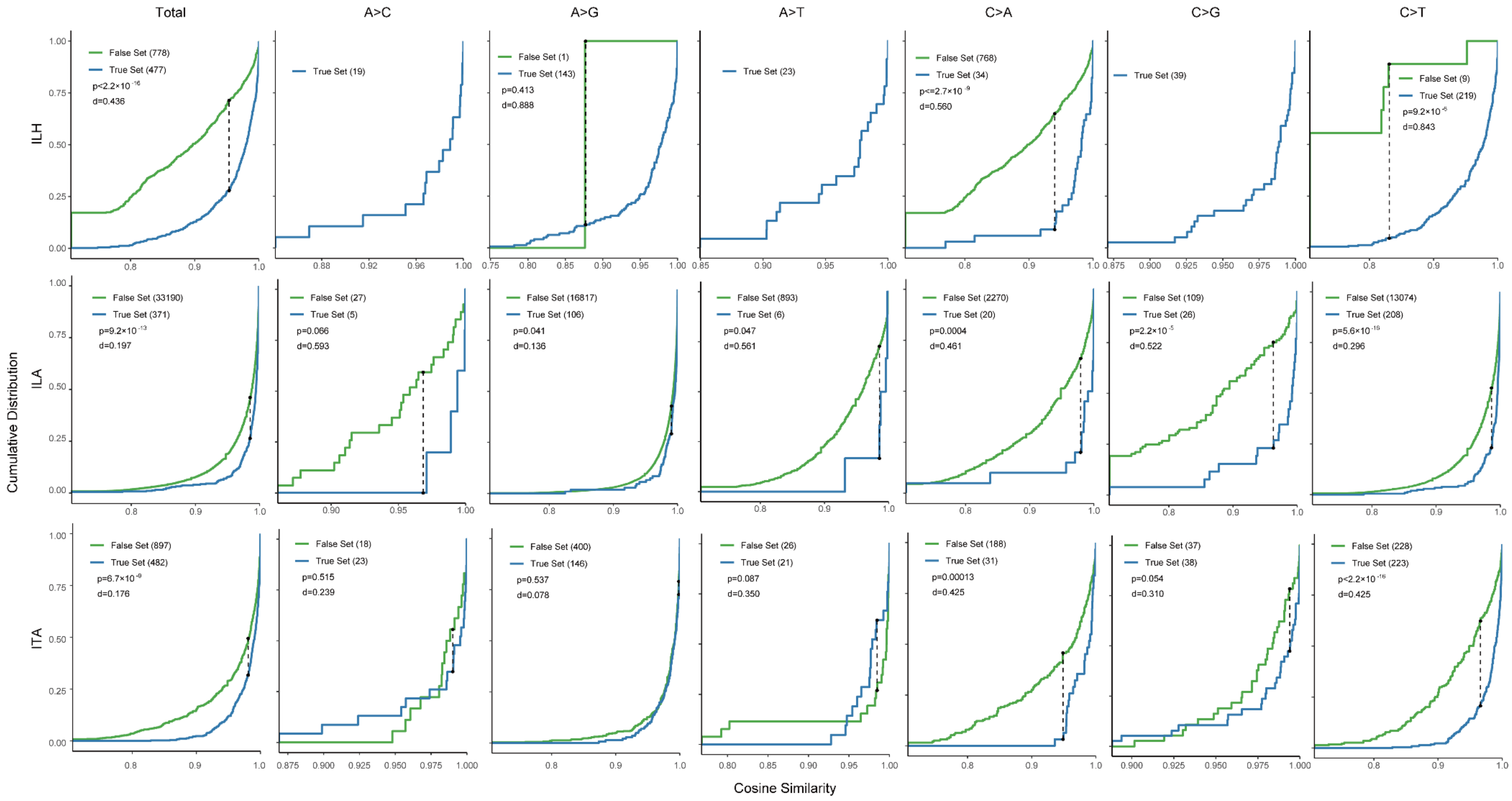

Supplementary Figure 9. Comparison of VAF concordance between true and false positive calls. For each called candidate (each dot in Fig. 3b), cosine similarity from y=x line was calculated for the VAF concordance measure between replicates, not to be affected by the absolute value of VAF. Distribution of cosine similarities are statistically compared between true and false positives using Kolmogorov–Smirnov (KS) test.

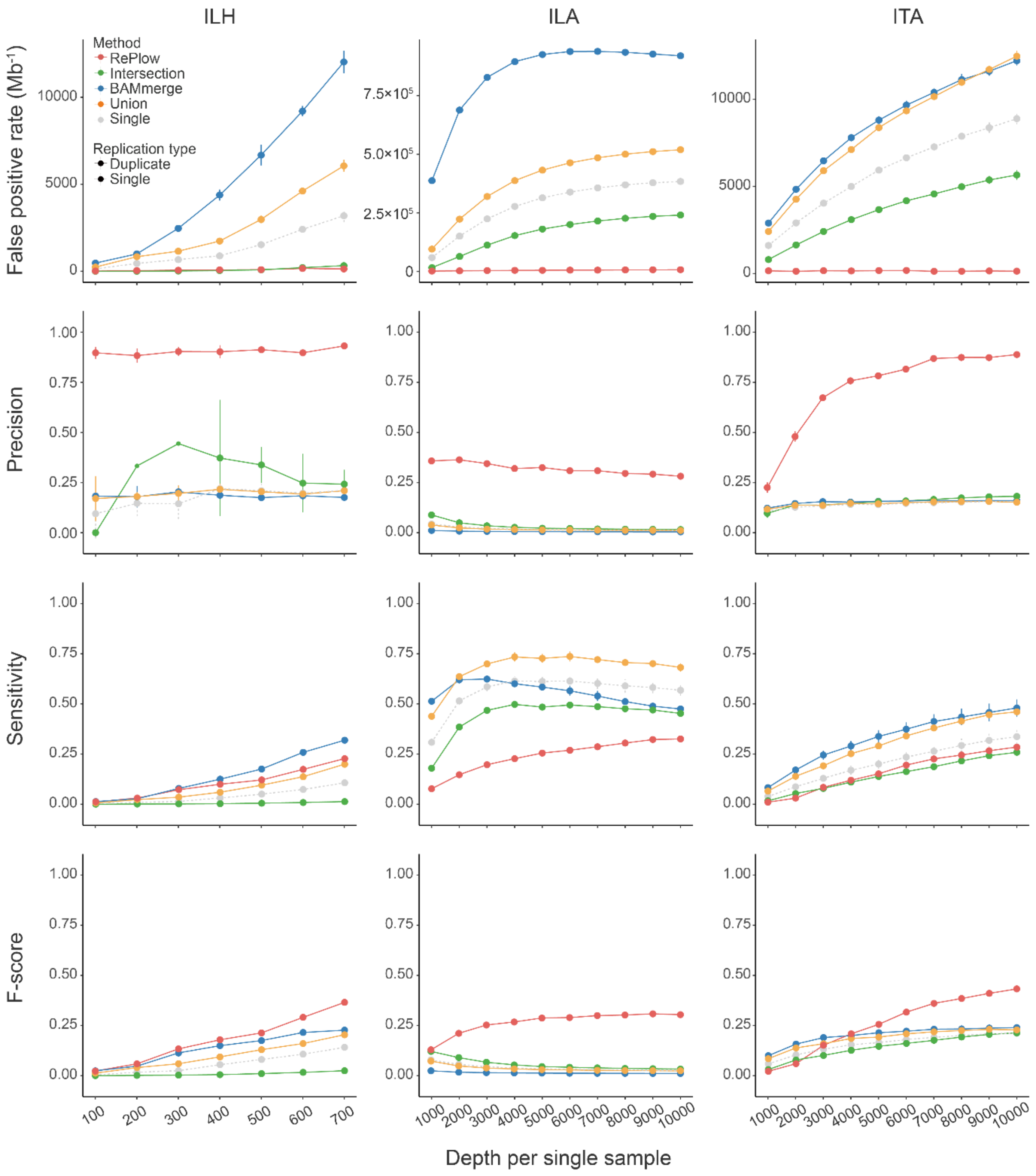

Supplementary Figure 10. Performance assessment with the library duplicates of test-base data. FPR, precision, sensitivity, and F-score were measured for sample A (0.5% VAF). All three combinations of duplicates were tested and their average performances were reported with 95% confidence interval. (typically smaller than marks). Source data are provided as a Source Data file.

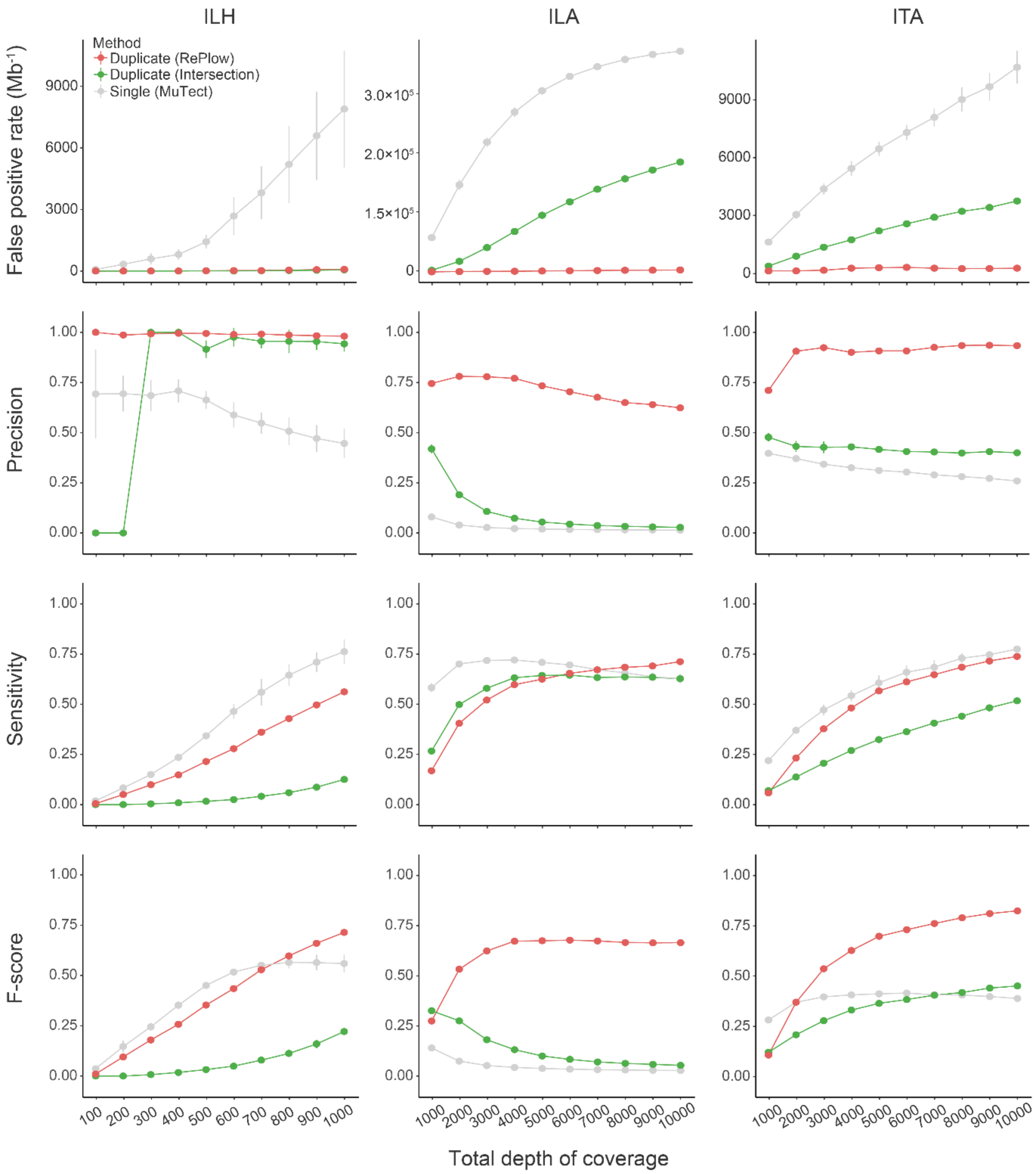

Supplementary Figure 11. Performance assessment with the single and duplicate libraries for the same depth of coverage. The depth of coverage for each replicate in the duplicate methods (RePlow, intersection) is exactly a half of a single library. For example, the results of RePlow at 1,000x are made from two 500x replicates. FPR, precision, sensitivity, and F-score were measured for sample B (1% VAF). All three combinations of duplicates were tested and their average performances were reported with 95% confidence interval. (typically smaller than marks). Source data are provided as a Source Data file.

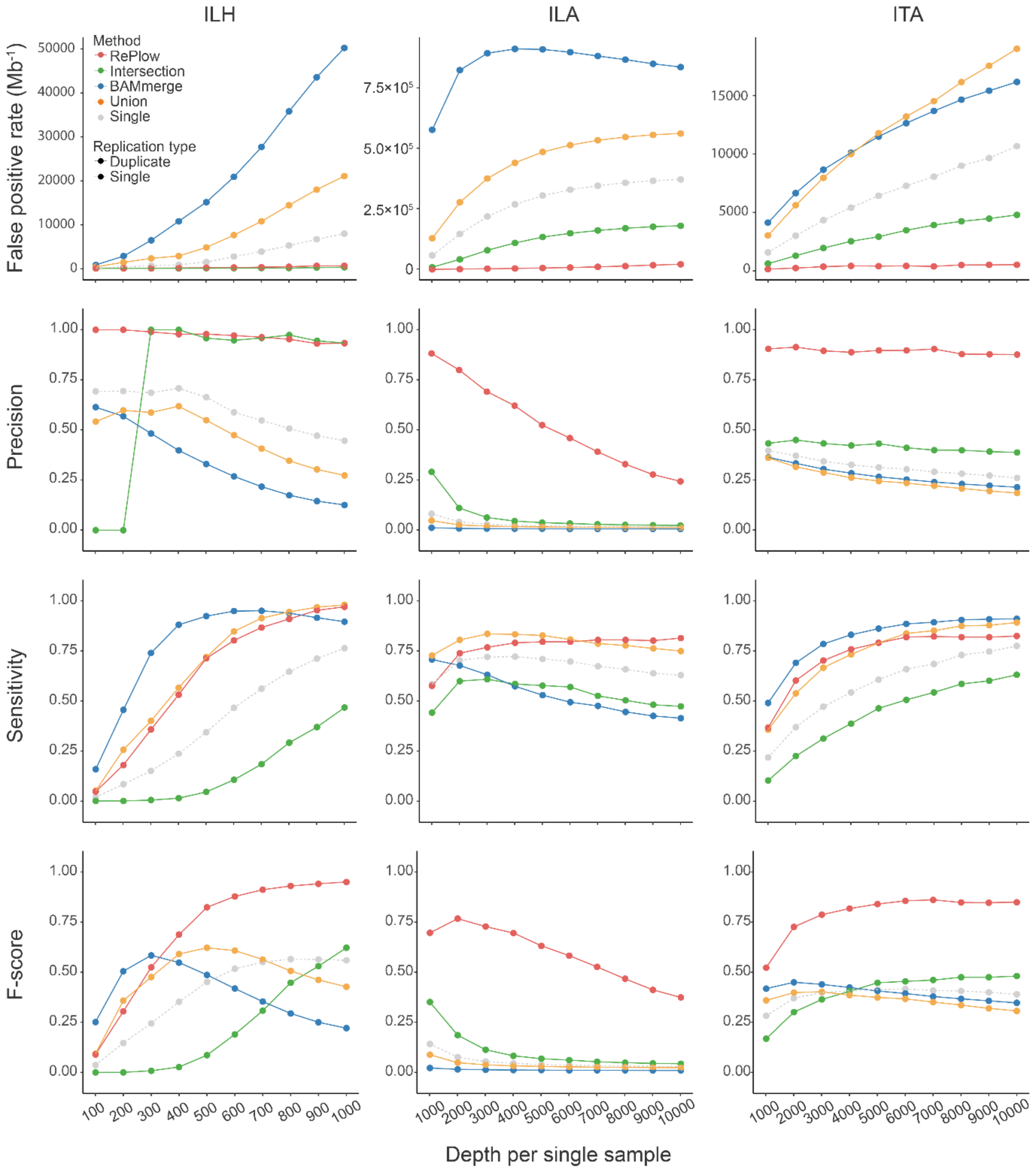

Supplementary Figure 12. Performance assessment with the library triplicates of test-base data. FPR, precision, sensitivity, and F-score were measured for sample B (1% VAF). With triplicates, RePlow demonstrated higher sensitivity than duplicates but showed decrease of precision in ILA datasets due to the increased number of overlapping errors which induce the over-estimation of concordance between replicates. Source data are provided as a Source Data file.

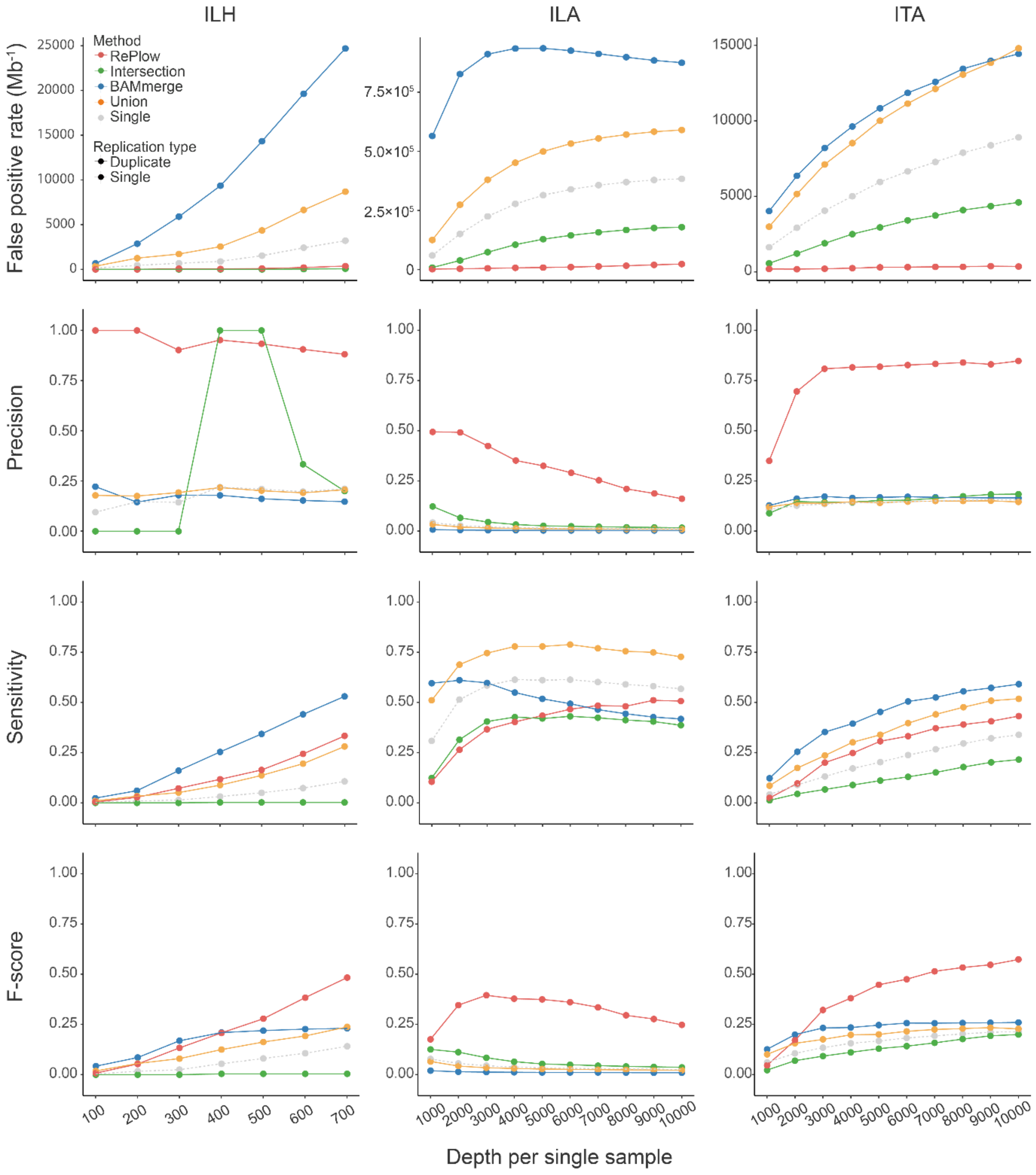

Supplementary Figure 13. Performance assessment with the library triplicates of test-base data. FPR, precision, sensitivity, and F-score were measured for sample A (0.5% VAF). With triplicates, RePlow demonstrated higher sensitivity than duplicates but showed decrease of precision in ILA datasets due to the increased number of overlapping errors which induce the over-estimation of concordance between replicates. Source data are provided as a Source Data file.

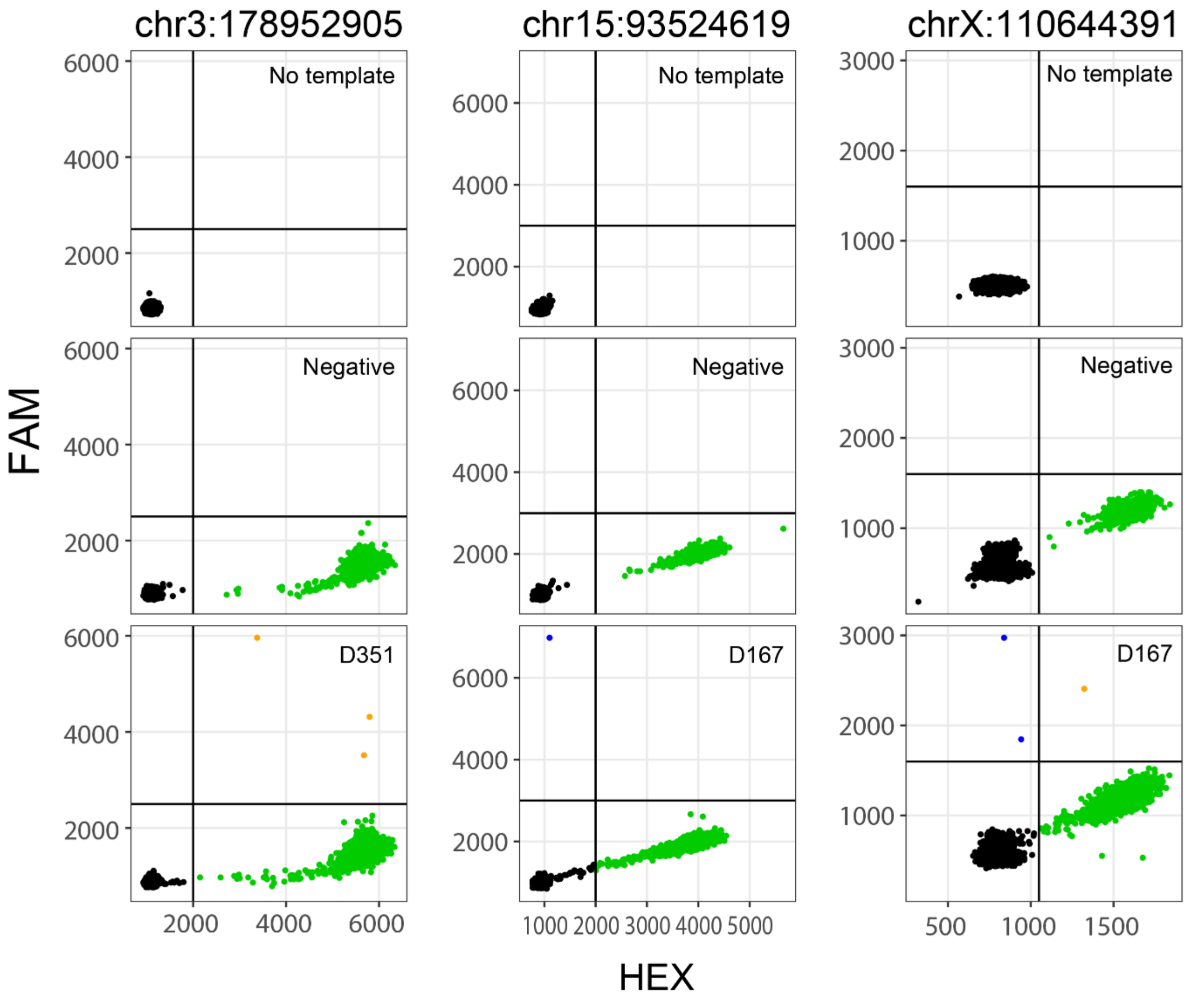

Supplementary Figure 14. Experimental validation of low-level mutations from the samples negative for pathogenic mutations in previous analysis. Among the seven low-VAF mutation candidates called by RePlow, two functional (missense) mutations (Fig. 4d) and three silent mutations were validated by droplet digital PCR. Designing PCR primers for the remaining two candidates failed.

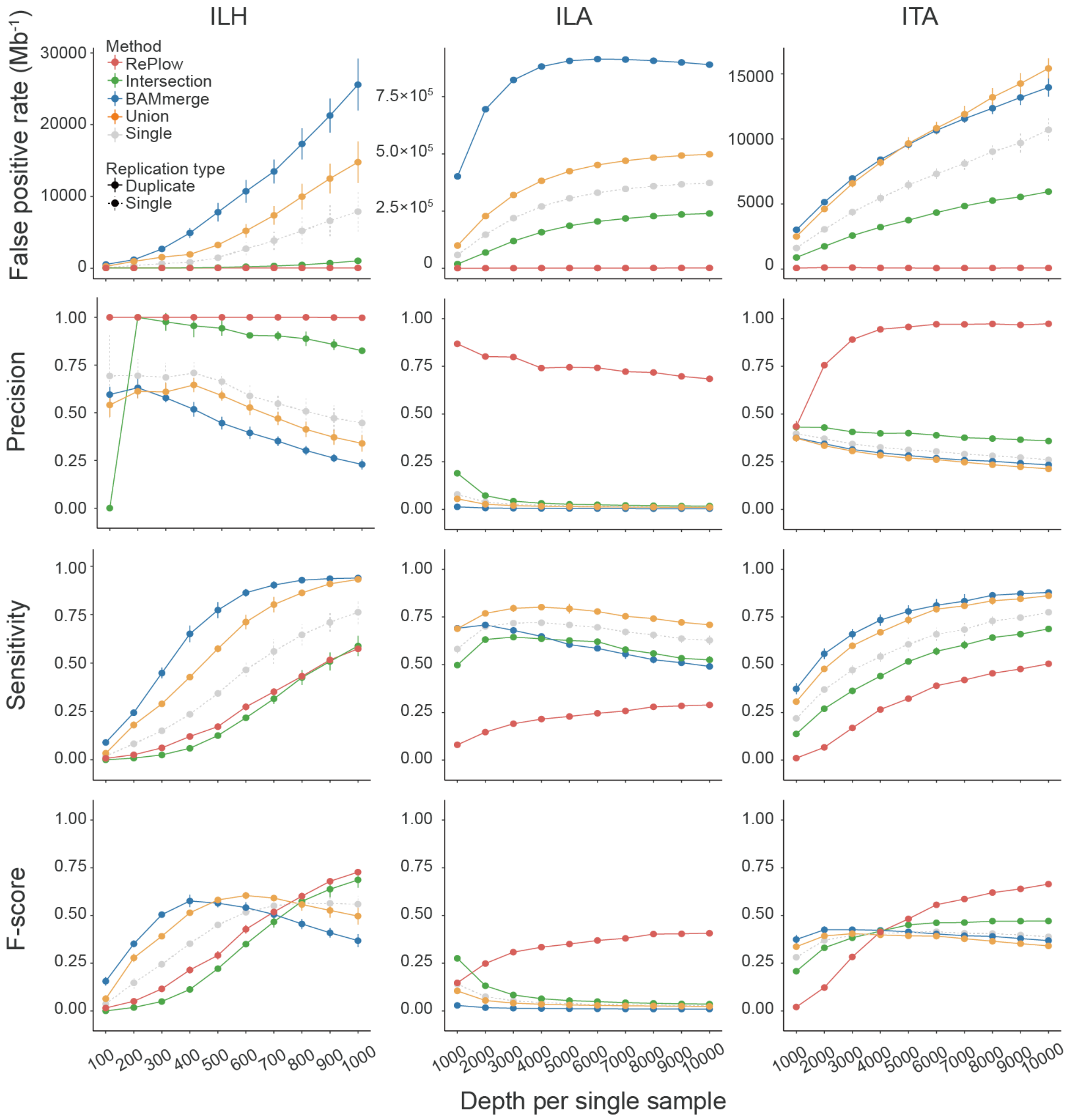

Supplementary Figure 15. Performance assessment of RePlow with the typical mutation rate (3×10^{-6}) for the library duplicates of sample B (1% VAF). Since excessive number of true mutations was prepared in the test-base data compared to the typical mutation rate, application of the default value to RePlow severely underestimated the amount of true variant and resulted in a significantly decreased sensitivity. Nevertheless, RePlow demonstrated the best overall performance (F-score) with the typical mutation rate based on the extraordinarily low number of false positive calls. All three combinations of duplicates were tested and their average performances were reported with 95% confidence interval. (typically smaller than marks). Source data are provided as a Source Data file.

Supplementary Tables

Supplementary Table 1. Summary of the test-base sequencing data and read-depth information

Platform	Abbr.	Sequencer	Read length (bp)	Target size (bp)	Covered true variant count	Average on-target depth (x)											
						A11	A12	A21	A31	B11	B12	B21	B31	C11	C12	D11	D12
Illumina hybrid-capture	ILH	HiSeq 2000	101x2	63,729	513	740.4	370.8	664.2	858.1	964.9	585.7	1,044.6	1,942.4	1,585.0	1,539.7	1,256.2	1,254.8
Illumina amplicon	ILA	HiSeq 2500	151x2	62,772	540	43,530.0	24,486.0	38,689.7	40,642.8	27,660.9	27,989.1	27,347.1	25,048.4	43,230.8	27,266.1	41,842.7	25,237.3
Ion Torrent amplicon	ITA	Ion Proton	125-275	122,311	591	12,500.3	12,395.1	10,580.0	12,362.5	10,367.5	7,926.7	12,327.3	11,089.1	7,777.7	8,908.4	8,940.1	10,208.8

Supplementary Table 2. Summary of the reference standard sequencing data and read-depth information

Platform	Cancer panel	Sequencer	Read length (bp)	Target size (bp)	Covered true variant count	Average on-target depth (x)		
						Rep1	Rep2	Rep3
ILH	SureSelect custom panel	HiSeq 2000	101x2	466,634	35	2,196.8	795.6	2,194.4
ITA	Ion AmpliSeq cancer hotspot panel v2	Ion Proton	111-187	22,027	35	55,785.8	40,902.5	61,467.7

Supplementary References

1. Li, H. & Durbin, R. Fast and accurate short read alignment with Burrows-Wheeler transform. Bioinformatics 25, 1754-60 (2009).
2. McKenna, A. et al. The Genome Analysis Toolkit: a MapReduce framework for analyzing next-generation DNA sequencing data. Genome Res 20, 1297-303 (2010).
3. Zhu, P. et al. OTG-snpcaller: an optimized pipeline based on TMAP and GATK for SNP calling from ion torrent data. PLoS One 9, e97507 (2014).
4. Cibulskis, K. et al. Sensitive detection of somatic point mutations in impure and heterogeneous cancer samples. Nat Biotechnol 31, 213-9 (2013).
5. Koboldt, D.C. et al. VarScan 2: Somatic mutation and copy number alteration discovery in cancer by exome sequencing. Genome Research 22, 568-576 (2012).
6. Saunders, C.T. et al. Strelka: accurate somatic small-variant calling from sequenced tumor–normal sample pairs. Bioinformatics 28, 1811-1817 (2012).
7. Lai, Z. et al. VarDict: a novel and versatile variant caller for next-generation sequencing in cancer research. Nucleic Acids Res 44, e108 (2016).
8. Kim, S. et al. Virmid: accurate detection of somatic mutations with sample impurity inference. Genome Biol 14, R90 (2013).
9. Wilm, A. et al. LoFreq: a sequence-quality aware, ultra-sensitive variant caller for uncovering cell-population heterogeneity from high-throughput sequencing datasets. Nucleic Acids Res 40, 11189-201 (2012).
10. Gerstung, M. et al. Reliable detection of subclonal single-nucleotide variants in tumour cell populations. Nat Commun 3, 811 (2012).
11. Chen, L., Liu, P., Evans, T.C., Jr. & Ettwiller, L.M. DNA damage is a pervasive cause of sequencing errors, directly confounding variant identification. Science 355, 752-756 (2017).
12. Costello, M. et al. Discovery and characterization of artifactual mutations in deep coverage targeted capture sequencing data due to oxidative DNA damage during sample preparation. Nucleic Acids Res 41, e67 (2013).
