# Supplementary material for: The use of technical replication for detection of low-level somatic mutations in next-generation sequencing
Source: Nat Commun. 2019 Mar 5;10:1047. doi: 10.1038/s41467-019-09026-y (PMC6400950; doi:10.1038/s41467-019-09026-y)
Supplement: Supplementary file 2 — Description of Additional Supplementary Files [file 41467_2019_9026_MOESM2_ESM.pdf]

Description of Additional Supplementary Files

File Name: Supplementary Data 1

Description: Designed target regions per sequencing platform for the analysis of test-base genome

File Name: Supplementary Data 2

Description: Platform-specific answer sets of test-base genome

File Name: Supplementary Data 3

Description: Target regions of two cancer panels for the analysis of reference standard

File Name: Supplementary Data 4

Description: Variant information of the reference standard and observed allele frequencies in the sequencing data
