# Supplementary material for: The use of technical replication for detection of low-level somatic mutations in next-generation sequencing
Source: Nat Commun. 2019 Mar 5;10:1047. doi: 10.1038/s41467-019-09026-y (PMC6400950; doi:10.1038/s41467-019-09026-y)
Supplement: Supplementary file 8 — Reporting Summary [file 41467_2019_9026_MOESM8_ESM.pdf]

nature research

Corresponding author(s): Sangwoo Kim

Last updated by author(s): Jan 31, 2019

Reporting Summary

Nature Research wishes to improve the reproducibility of the work that we publish. This form provides structure for consistency and transparency in reporting. For further information on Nature Research policies, see Authors & Referees and the Editorial Policy Checklist.

Statistics

For all statistical analyses, confirm that the following items are present in the figure legend, table legend, main text, or Methods section.

n/a	Confirmed	
☐	☒	The exact sample size (n) for each experimental group/condition, given as a discrete number and unit of measurement
☐	☒	A statement on whether measurements were taken from distinct samples or whether the same sample was measured repeatedly
☐	☒	The statistical test(s) used AND whether they are one- or two-sided<br>*Only common tests should be described solely by name; describe more complex techniques in the Methods section.*
☐	☒	A description of all covariates tested
☐	☒	A description of any assumptions or corrections, such as tests of normality and adjustment for multiple comparisons
☐	☒	A full description of the statistical parameters including central tendency (e.g. means) or other basic estimates (e.g. regression coefficient) AND variation (e.g. standard deviation) or associated estimates of uncertainty (e.g. confidence intervals)
☒	☐	For null hypothesis testing, the test statistic (e.g. F, t, r) with confidence intervals, effect sizes, degrees of freedom and P value noted<br>*Give P values as exact values whenever suitable.*
☐	☒	For Bayesian analysis, information on the choice of priors and Markov chain Monte Carlo settings
☒	☐	For hierarchical and complex designs, identification of the appropriate level for tests and full reporting of outcomes
☒	☐	Estimates of effect sizes (e.g. Cohen's d, Pearson's r), indicating how they were calculated

Our web collection on statistics for biologists contains articles on many of the points above.

Software and code

Policy information about availability of computer code

Data collection	No software was used for data collection
Data analysis	The implemented program of our algorithm (v1.1) is available in the designated open source repository (SourceForge) https://sourceforge.net/projects/replow/

For manuscripts utilizing custom algorithms or software that are central to the research but not yet described in published literature, software must be made available to editors/reviewers. We strongly encourage code deposition in a community repository (e.g. GitHub). See the Nature Research guidelines for submitting code & software for further information.

Data

Policy information about availability of data

All manuscripts must include a data availability statement. This statement should provide the following information, where applicable:

- Accession codes, unique identifiers, or web links for publicly available datasets
- A list of figures that have associated raw data
- A description of any restrictions on data availability

The raw NGS data of the test-base (39 samples, used for Figure 1A, 2C, 3B and 4A) and the independent cancer panel sequencing data (8 samples, Figure 4C) have been deposited in the NCBI Sequence Read Archive with the accession code SRP183061 [https://www.ncbi.nlm.nih.gov/sra/?term=SRP183061].

Field-specific reporting

Please select the one below that is the best fit for your research. If you are not sure, read the appropriate sections before making your selection.

☒ Life sciences ☐ Behavioural & social sciences ☐ Ecological, evolutionary & environmental sciences

For a reference copy of the document with all sections, see nature.com/documents/nr-reporting-summary-flat.pdf

Life sciences study design

All studies must disclose on these points even when the disclosure is negative.

Sample size	We included a total of 39 samples that consists of 4 spike-in samples and 3 matched controls in 3 distinct platforms including 6 triplicates of library replication and 6 duplicate of sequencing replication. (Figure 1, Methods paragraph 1 and 2)
Data exclusions	In the validation of brain tissue sequencing, three samples were selected based on three criteria: 1) fresh frozen brain tissue samples are available, 2) matched control samples are available and 3) no pathogenic mutations were identified in the previous investigations. (Variant detection with RePlow in Results, paragraph 4)
Replication	All replicates from sequencing and library replication were tested to measure the performance of previous and developed methods and obtained concordant results between replicates.
Randomization	N/A (no randomization is necessary)
Blinding	N/A

Reporting for specific materials, systems and methods

We require information from authors about some types of materials, experimental systems and methods used in many studies. Here, indicate whether each material, system or method listed is relevant to your study. If you are not sure if a list item applies to your research, read the appropriate section before selecting a response.

Materials & experimental systems

n/a	Involved in the study	
☒	☐	Antibodies
☒	☐	Eukaryotic cell lines
☒	☐	Palaeontology
☒	☐	Animals and other organisms
☒	☐	Human research participants
☒	☐	Clinical data

Methods

n/a	Involved in the study	
☒	☐	ChIP-seq
☒	☐	Flow cytometry
☒	☐	MRI-based neuroimaging
